# *In vivo* therapeutic potential of Dicer-hunting siRNAs targeting infectious hepatitis C virus.

**DOI:** 10.1038/srep04750

**Published:** 2014-04-23

**Authors:** Tsunamasa Watanabe, Hiroto Hatakeyama, Chiho Matsuda-Yasui, Yusuke Sato, Masayuki Sudoh, Asako Takagi, Yuichi Hirata, Takahiro Ohtsuki, Masaaki Arai, Kazuaki Inoue, Hideyoshi Harashima, Michinori Kohara

**Affiliations:** 1 Department of Microbiology and Cell Biology, Tokyo Metropolitan Institute of Medical Science, Tokyo 156-8506, Japan; 2 Division of Gastroenterology, Showa University Fujigaoka Hospital, Yokohama, Japan; 3 Laboratory of Innovative Nanomedicine, Faculty of Pharmaceutical Sciences, Hokkaido University, Hokkaido 060-0812, Japan; 4 Kamakura Research Laboratories, Chugai Pharmaceutical Co., Ltd., Kamakura, Kanagawa 247-8530, Japan; 5 Advanced Medical Research Laboratory, Mitsubishi Tanabe Pharma Corporation, 1000, Kamoshida-cho, Aoba-ku, Yokohama 227-0033, Japan; 6 Present address, Department of Virology & Liver Unit, Nagoya City University Graduate School of Medical Sciences, Kawasumi, Mizuho, Nagoya 467-8601, Japan; 7 These authors contributed equally to this work.

## Abstract

The development of RNA interference (RNAi)-based therapy faces two major obstacles: selecting small interfering RNA (siRNA) sequences with strong activity, and identifying a carrier that allows efficient delivery to target organs. Additionally, conservative region at nucleotide level must be targeted for RNAi in applying to virus because hepatitis C virus (HCV) could escape from therapeutic pressure with genome mutations. *In vitro* preparation of Dicer-generated siRNAs targeting a conserved, highly ordered HCV 5′ untranslated region are capable of inducing strong RNAi activity. By dissecting the 5′-end of an RNAi-mediated cleavage site in the HCV genome, we identified potent siRNA sequences, which we designate as Dicer-hunting siRNAs (dh-siRNAs). Furthermore, formulation of the dh-siRNAs in an optimized multifunctional envelope-type nano device inhibited ongoing infectious HCV replication in human hepatocytes *in vivo*. Our efforts using both identification of optimal siRNA sequences and delivery to human hepatocytes suggest therapeutic potential of siRNA for a virus.

Hepatitis C virus (HCV) is a major etiological agent that causes chronic hepatitis, liver cirrhosis, and hepatocellular carcinoma. Despite clinical improvements provided by combination therapy with interferon-alpha and ribavirin, this therapeutic approach fails in about half of the patients^1^. Clinical proof-of-concept studies for new therapeutic agents have been reported, and several compounds have progressed into preclinical studies^2^,^3^. However, drug-resistant viruses appear to emerge readily in response to pharmacological selection by novel protease and RNA polymerase inhibitors. Therefore, the development of new therapies for refractory and diverse HCV genotypes represents a major public health objective.

RNA interference (RNAi) results from sequence-specific post-transcriptional gene silencing by double-stranded RNA^4^,^5^. The effectors of RNAi are short interfering RNA (siRNA) duplexes (~21–23 nt), which play a key role in the specific degradation of target mRNA. Recent studies have shown that a critical challenge in therapeutic application of RNAi is the identification of potent siRNAs; the functionality of these molecules is affected by the duplex nucleotide base preference and the accessibility of the target RNA^6^,^7^,^8^,^9^,^10^. Additionally, in the treatment of HCV, the emergence of “escaped/resistant” viruses that harbor point mutations in the target region is a major concern in the potential clinical application of RNAi^11^,^12^,^13^. Although current algorithms for the selection of anti-viral siRNAs consist of the guidelines previously derived for conserved sequences of viral genomes^14^, there is compelling evidence that one siRNA-resistant human immunodeficiency virus (HIV)-1 had no mutations in the target site of the mRNA, but instead contained a point mutation 7 nt upstream of the target site^15^. This observation means that non-target mutations altering the local RNA secondary structure could ablate the RNAi activity without mutation in the siRNA target sequence. Additionally, there are reports that the secondary structure of target sites in mRNAs strongly reduce siRNA-mediated RNAi activity^16^,^17^, hence the accessibility of certain local target structures on the mRNA is an important determinant in the gene silencing ability of siRNAs^18^. Therefore, the prediction of an effective siRNA targeting to virus genome cannot be identified in the same fashion. Here, we attempt to predict active siRNA sequences using Dicer recognition in a conserved and highly ordered region of the HCV genome.

Separately, delivering siRNAs to intracellular targets also remains a major obstacle. Over the last several years, significant efforts have been devoted to exploring novel delivery strategies; examples include cationic liposomes and polymer-based nanocarriers^19^. To overcome the problems associated with *in vivo* delivery of siRNAs, both biodistribution to the target organ and intracellular trafficking in target cells of nanocarriers need to be addressed. High siRNA-encapsulation efficacy and uniform particle size also are required. We describe here an improved delivery system consisting of a multifunctional envelope-type nano device (MEND)^20^, in which siRNA is encapsulated by cationic charged lipid envelope. To avoid the undesired interaction of cationic MENDs with biological components and subsequent loss of activity, a pH-sensitive property was incorporated into the lipid envelope of MEND by using a novel pH-sensitive cationic lipid, YSK05^21^. For enhanced delivery of cargos into cells, pH-sensitive liposomes have been investigated since the mid-1980s^22^,^23^. Recently, significant progress has been made in *in vivo* systemic siRNA delivery with lipid nanoparticles (LNPs) composed of ionizable cationic lipids. These LNPs represent neutral surface at physiological pH, but convert to a cationic form under acidic conditions (as expected in the endosome); siRNAs delivered by this mechanism provide efficient reduction of target gene expression in liver^24^. In this study, we describe the development of liver-targeted MENDs containing YSK05 for delivery of the active siRNAs, a system with therapeutic potential for the treatment of HCV-infected liver.

## Results

### Dicer-hunting siRNA targeting the HCV IRES has powerful silencing efficacy

**Figure 1:**
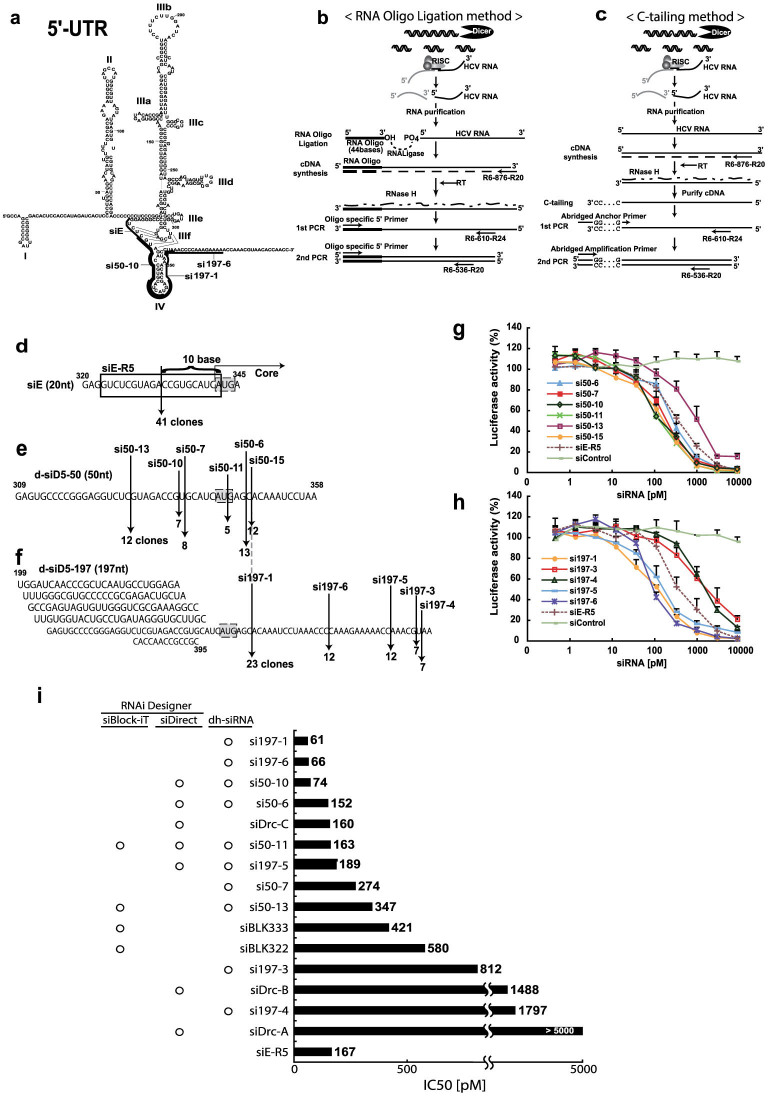
Generation and efficacy of dicer-hunting siRNAs (dh-siRNAs). (a) Complicated secondary structure of the 5′-UTR in the HCV genome^50^ and representation of the potent siRNA sequences we identified (si50-10, si197-1, and si197-6). (b, c) 5′-RACE strategy for identifying the RNAi cleavage sites of the target HCV genome using the RNA oligo ligation method (b) and the C-tailing at the 3′-end of RNA method (c). (d–f) Representation of the cleavage sites by siRNA and the number of clones in the sequences of HCV RNA genome that were used as templates. Cleavage site of the HCV RNA genome by siE (d), d-siD5-50 (e), and d-siD5-197 (f) was identified using the two RACE methods (2b and 2C). (g, h) Evaluation of silencing efficacy of dh-siRNAs. The HCV-replicon cells with reporter genes were transfected with the dh-si50 series (si50-6, 7, 10, 11, 13, and 15) (g) or the dh-si197 series (si197-1, 3, 4, 5, and 6) derived from d-si197 (h). Luciferase activity was measured after 48 hr. Data are presented as mean ± s.d. (*n* = 5) of values normalized to those obtained with mock-transfected cells. (i) Comparison of IC50 for inhibition of HCV replication by siRNAs that were derived from dh-siRNA or predicted by siRNA web design tools. Based on the HCR6 (genotype 1b) sequence, commercial software (siBlock-iT, siDirect) predicted several siRNA sequences (Supplementary table 1). IC50 values represent the mean for independent determinations (*n* = 5) using HCV replicon cells harboring subgenomic HCR6 sequences.

The most conserved sequences among different HCV genotypes are 5′ untranslated region (UTR). The HCV 5′ UTR forms an RNA folded structure which has functional for the internal ribosome entry site (IRES)^25^, and then allows protein synthesis to proceed in a cap-independent manner, implying an important role in key step of the viral translation and replication. Therefore, siRNAs targeting the IRES are expected to reduce the chances of viral mutational escape because the conserved 5′ UTR are likely to contain both structurally and functionally constrained elements (Fig. 1a). As the HCV IRES has local higher order structures at the RNA level, random sequence of siRNA targeting to the region might not exclusively induce RNAi activity. To identify an effective siRNA targeting the IRES sequences, we previously tested the efficacy of several synthetic siRNAs using an *in vitro* HCV-replicon assay, revealing that the siE sequences had an IC50 of 167 pM in this assay^26^. In addition, we found that the Dicer-generated siRNAs (d-siRNAs) targeting in the IRES not only provided silencing for heterogeneous target mRNA, but also exhibited even stronger silencing for homogeneous target HCV RNA^26^ (supplementary Fig.1). Thus, we suspected that d-siRNAs contain powerful siRNA sequences, and/or that d-siRNAs, comprised of a library of several siRNAs, are additive for silencing activity. Previous work has shown that dsRNAs that are longer than 21-mer siRNAs (e.g., 27-mer dsRNAs^27^ or 29-mer shRNAs^28^) display enhanced potency in RNAi. We speculated that these longer dsRNAs serve as substrates for the Dicer endonuclease, directly linking the production of siRNA to incorporation into the RNA-induced silencing complex (RISC), for example via the RISC-loading complex^29^. Therefore, we sought to identify the active siRNA sequences in a library of d-siRNAs. Specifically, we screened an siRNA cleavage site of the target HCV genome by using two distinct 5′-rapid amplification of cDNA ends (RACE) methods. The first RACE method employed RNA oligo ligation, such that the 5′-end of a HCV RNA (following cleavage by RISC) was ligated to an RNA oligomer (44 bases); the resulting molecule then was subjected to cDNA synthesis, nested-PCR amplification, and sequencing (Fig. 1b). The second RACE method employed C-tailing, such that a series of C nucleotides were attached at the 3′-end of the synthetic cDNA; the resulting molecule was then annealed with an abridged anchor primer and subjected to nested-PCR amplification and sequencing (Fig. 1c). We first validated the ability of these RACE methods to detect siRNA-mediated cleavage. Synthetic siE was transfected into REF cells^30^ (which harbor the divided-full genome replicon), and total RNA was purified from the transfected cells. Using the two independent 5′-RACE methods, we demonstrated that a unique site, at the 5′-end of the HCV genome, was cleaved following treatment with the siE (Fig. 1d). The cleavage site on the target HCV RNA genome was located 10 nt downstream of the 5′-end of the guide siE in 41 of 48 clones. Previous reports show that RISC cleaves the mRNA at a site precisely 10 or 11 nt downstream of the 5′ end of the siRNA guide strand^31^. Thus, our results confirm the utility of RACE for identifying the 5′-end of the cleaved target mRNA during silencing. Next, we determined the 5′-end of HCV genome cleaved by the d-siRNAs, which consist of several kinds of siRNA products generated *in vitro* by activity of the Dicer endonuclease. Treatment with the d-siD5-50 yielded six separate cleavage sites across the 50-nt-long target region of the HCV RNA genome (Fig. 1e). Treatment with the d-siD5-197 yielded five separate cleavage sites across the 197-nt-long target region of the HCV RNA genome (Fig. 1f). Notably, the si197-1 site, which was observed among the majority of d-siD5-197 clones, was identical to the si50-15 site. Additionally, note that we could not detect the HCV-specific siE site in cloning of d-siD5-50 and d-siD5-197 cleavage sites, although the siE site had exhibited superior silencing efficacy in previous studies^26^.

siRNAs, which consist of duplexes of 21-nt RNAs that are base-paired with 2-nt 3′ overhangs, were designed based on the cleavage site defined by d-siRNAs; these siRNAs therefore were designated as Dicer-hunting siRNAs (dh-siRNA). We synthesized a series of 10 individual dh-siRNAs and transfected each into R6FLR-N replicon cells having with the reporter gene (Fig. 1g and 1h). Compared to the efficacy of the siE, several of these individual dh-siRNAs showed strong silencing activity against HCV replication. The efficacy of the si197-1 was three times higher than that of siE; the other two dh-siRNAs silenced HCV replication with efficacy two times higher than that of siE.

Currently, software for efficient siRNA design of antiviral RNAi are available (siVirus^14^, siDirect^32^, Block-iT (Invitrogen), and the web-based antiviral siRNA design software). These programs make prediction based on highly conserved regions of divergent viral sequences, and claim to minimize off-target effects that might result from effects on the molecular mechanism of RISC assembly or from the sequence preferences of the RISC endonuclease. In the HCV RNA genome sequences, we predicted an effective siRNA targeting the IRES (nt 199-395) using the three commercial software programs. We compared a software-proposed siRNA as previously reported with dh-siRNAs we report here. The values of IC50 for the predicted siRNAs in HCV-replicon assays are presented in Fig. 1i. In the IRES region, the siBlock-iT software proposed four kinds of siRNAs; siDirect proposed seven kinds of siRNAs; and siVirus proposed no siRNA. Five kinds of dh-siRNA were proposed by either siBlock-iT or siDirect software, and the dh-si50-11 was proposed by both siBlock-iT and siDirect software. However, the first- and second-most effective siRNAs, dh-si197-1 and dh-si197-6, were proposed by neither siBlock-iT nor siDirect software. These corresponded to molecules with IC50s of 61 and 66 picomolar (respectively) in our replicon assays (Fig. 1i). These results indicated that dh-siRNA prepared based on the cleavage site of the target mRNA treated with Dicer-generated siRNAs were powerful siRNA target sites; commercial design software programs were not of service for siRNA prediction in the HCV IRES region. In the case of siRNAs targeting endogenous mRNA, precise processing by Dicer is not critical because any cleavage frame would result in functional siRNAs; however the correlation of siRNA sequence with structurally/functionally constrained elements of HCV RNA is expected be critical for RNAi function.

### Combination of the Dicer-hunting siRNA leads to enhanced reduction for heterogeneous HCV RNA

**Figure 2:**
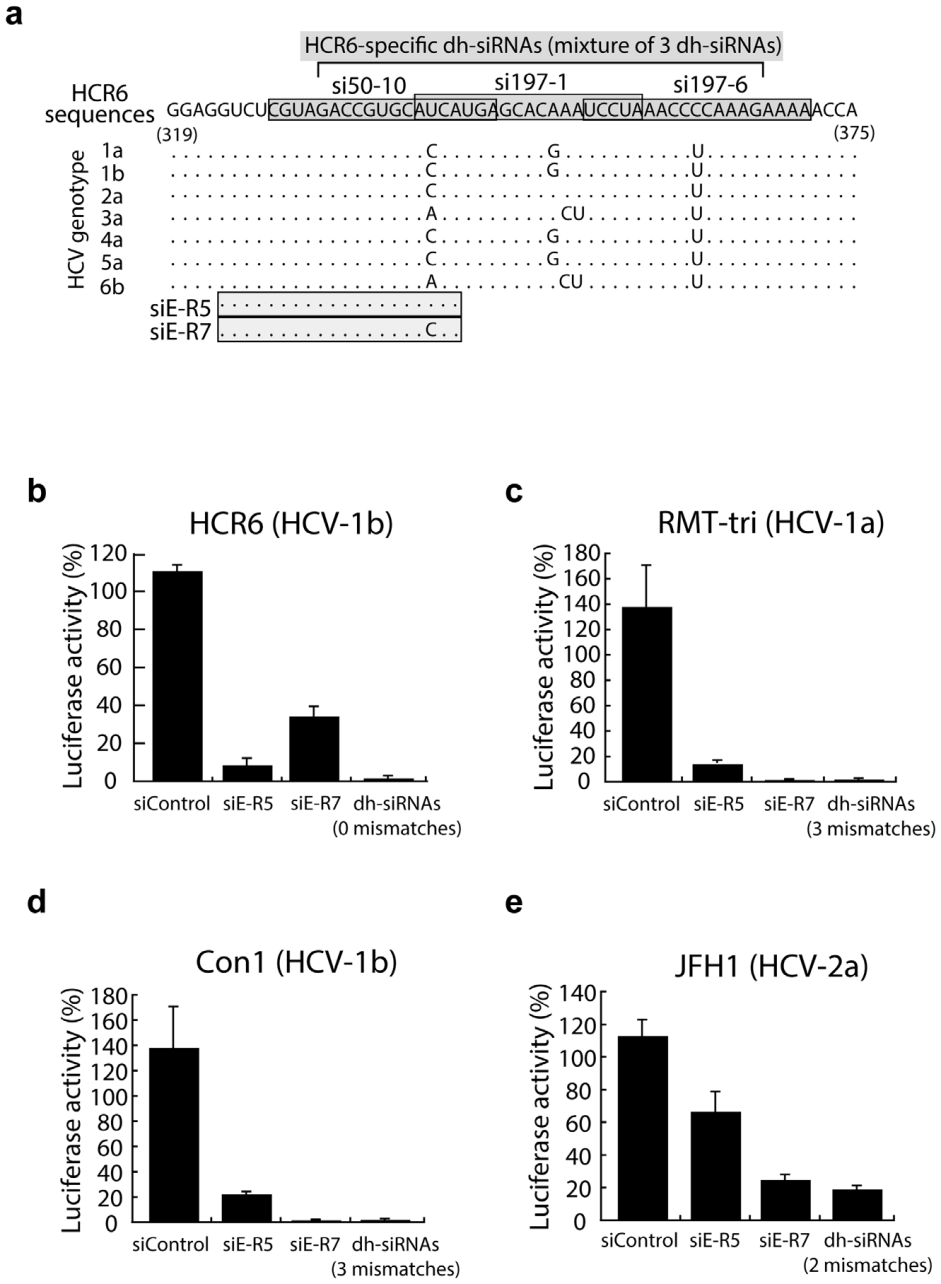
Efficacy against heterogeneous viruses by mixtures of dh-siRNAs. (a) Alignment of HCV genotype sequences in the region targeted by the dh-siRNAs and siRNA sequences. Aligned sequences for siRNAs are indicated by shaded boxes; dots indicate matches to the consensus (HCR6); individual bases indicate genotype-specific differences. (b–e) Inhibition efficacy by mixture of three HCR6-based dh-siRNAs against homogeneous replicon cells (b), heterogeneous replicon cells with RMT-tri sequences (genotype-1b; 3 mismatches) (c), heterogeneous replicon cells with Con1 sequences (genotype-1b; 3 mismatches) (d), and heterogeneous replicon cells with JFH1 sequences (genotype-2a; 2 mismatches) (e). All samples were assessed at 72 hr after transfection. Data are presented as mean ± s.d. (n = 5) of values normalized to those obtained with mock-transfected cells.

The notoriously error-prone replication of RNA viruses is a severe challenge for the development of siRNA-based anti-viral therapies. Indeed, HCV displays a high rate of mutation and is classified into distinct genotypes (1 to 6) (Fig. 2a). Therefore, to examine whether a mixture of dh-siRNAs can silence the replication of heterogeneous HCV RNA, we transfected HCR6 sequence-specific dh-siRNAs into heterogeneous replicon cells, specifically cells that harbor RMT-tri (genotype 1a), Con1 (genotype 1b), or JFH1 (genotype 2a) replicons (Fig. 2b–e). The combination treatment included si197-1, si197-6, and si50-10. All three of these sequences are HCR6-specific (genotype 1b), and the combination silenced the homogeneous HCV replication more effectively than the HCR6-specific siE-R5 alone (Fig. 2b). We next transfected a mixture of the dh-siRNAs into RMT-tri replicon cells, which harbor heterogeneous sequences compared to the HCR6 sequences. Treatment with the combination of three dh-siRNAs targeting the HCR6 sequences yielded a single mutation within the RMT-tri (genotype 1a) target sequences (Fig. 2a). The combination treatment (dh-si197-1, dh-si197-6, and dh-si50-10) represented a total of 3 mismatches versus the heterogeneous RMT-tri replicon but still provided silencing of heterogeneous RMT-tri replication. For comparison, siE-R5, which represents a single mismatch versus the RMT-tri target sequence, exhibited reduced silencing against the heterogeneous RMT-tri genome (Fig. 2c). Moreover, the replication of Con1 (another virus of genotype 1b) and JFH1 (a virus of genotype 2a) were silenced by the dh-siRNA combination treatment, with efficacy equal to or exceeding that of replicon-specific siE-R7 (Fig. 2d and 2e). These results indicate that combination treatment with dh-siRNAs targeting the IRES sequence can silence and reduce HCV replication, even when applied to heterogeneous HCV genomes.

### Characterization and optimization of pH-sensitive MENDs containing YSK05 for efficient siRNA delivery to hepatocytes

**Figure 3:**
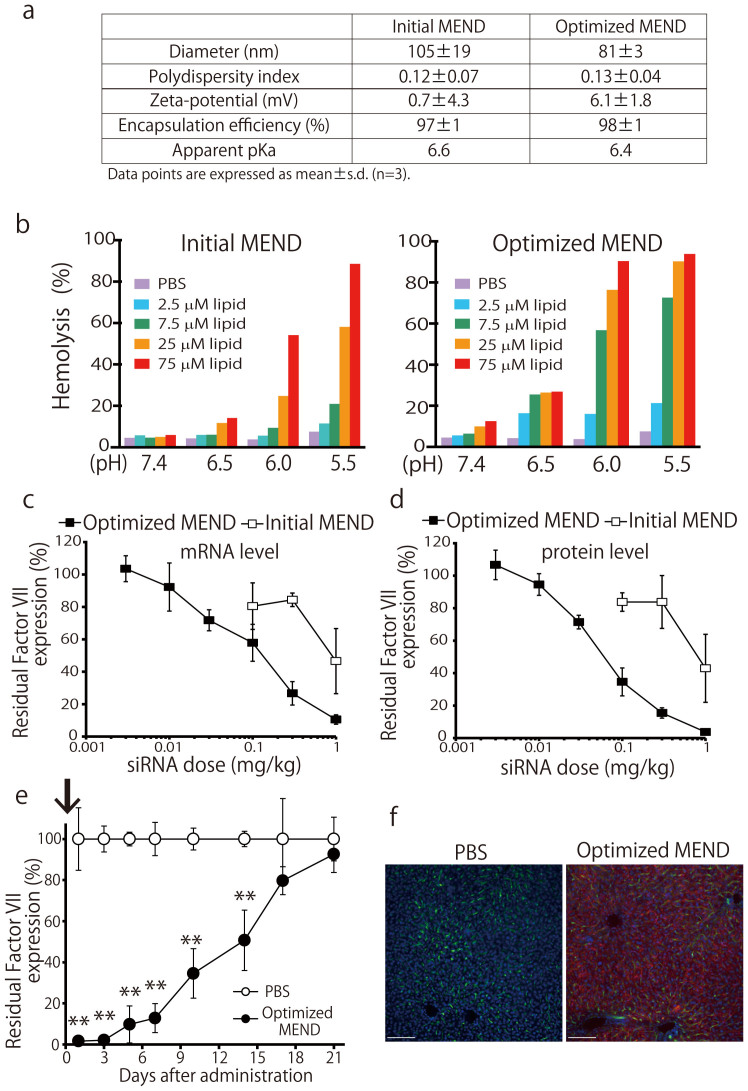
*In vitro* and *in vivo* characterization of the optimized MEND. (a) Physical properties of multifunctional envelope-type nano devices (MENDs). (b) The membrane fusion activity of initial and optimized MENDs was assessed by an *in vitro* hemolysis assay. Values (normalized to hemolysis with known lysing agent (Triton)) are presented as the mean (n = 3). N.C.; negative control (treated with PBS). (c, d) *In vivo* hepatic *Factor VII* mRNA (c) and serum Factor VII protein (d) levels at 48 hr after the administration of the initial and optimized MENDs. Data points are presented as the mean ± s.d. (n = 3) of values normalized to those obtained with uninjected mice. (e) *In vivo* persistence of knockdown was investigated by monitoring serum Factor VII levels at the indicated number of days after administration of optimized MENDs at a dose of 1.0 mg siRNA per kg. **P<0.01. Data points are presented as the mean ± s.d. (n = 3) of values normalized to those obtained with uninjected mice. (f) Liver tissues were collected after single injection of PBS or the optimized MENDs encapsulating Cy5-siRNA (red) and stained with FITC-isolectin B4 (green) and Hoechst33342 (blue) to detect blood vessels and nuclei (respectively). Bars represent 100 μm.

One of the challenges in developing RNAi therapeutics is the efficient delivery of siRNA into the cell affected by a given disease, for instance into the cytoplasm of HCV-infected hepatocytes. As a possible solution, we attempted dh-siRNAs delivery to hepatocytes using a pH-sensitive MEND containing YSK05, a pH-sensitive cationic lipid. The physical properties of siRNAs formulated in MENDs are shown in Fig. 3a. To examine the utility of MENDs containing YSK05 for the *in vivo* systemic delivery of siRNA to liver, mice were treated with siRNAs formulated in MENDs. We first screened the lipid composition to achieve efficient knockdown (supplementary Fig. 2). We found that MEND composed of YSK05, cholesterol, and PEG-DMG (at ratios of 70:30:3) provided the most efficient knockdown of target *srbI* gene in liver (supplementary Fig. 2b). Next, the membrane fusion activity of the optimized MEND was assessed by hemolysis assay^21^ at the indicated pH to evaluate the potency for escape from endosomes via membrane fusion (Fig. 3b). Membrane fusion activity for optimized MEND was increased compared to initial MEND at acidic pH. On the other hand, the apparent pKa of optimized MEND was comparable to that of the initial MEND (Fig. 3a). Factor VII is a secreted protein that can be readily measured in plasma, providing an index for the level of target gene knockdown in liver^24^,^33^. Therefore, we evaluated Factor VII knockdown using MENDs. As shown in Fig. 3c and 3d, initial and optimized MENDs decreased both liver mRNA and plasma level of Factor VII protein in a dose-dependent manner, with ED50s of approximately 0.8 mg/kg and 0.06 mg/kg (initial and optimized MENDs, respectively) for plasma levels of Factor VII. The results suggested that optimized MEND successfully induced the efficient knockdown in liver after systemic administration in mice. Next, the durability of knockdown of plasma Factor VII was determined. Single injection of optimized MEND was capable of mediating knockdown for at least 14 days at 50% inhibition (Fig. 3e). Finally, the distribution of siRNA was observed in the livers of animals treated with optimized MEND encapsulating Cy5-labeled siRNA. As shown in Fig 3f, MEND uniformly delivered siRNA to parenchymal cells in liver. These results suggested that optimized MENDs loaded with HCV-targeting dh-siRNAs can induce silencing against cytosolic replicating HCV. Therefore, further investigation was performed using the optimized MEND.

### Silencing infectious HCV *in vivo*

**Figure 4:**
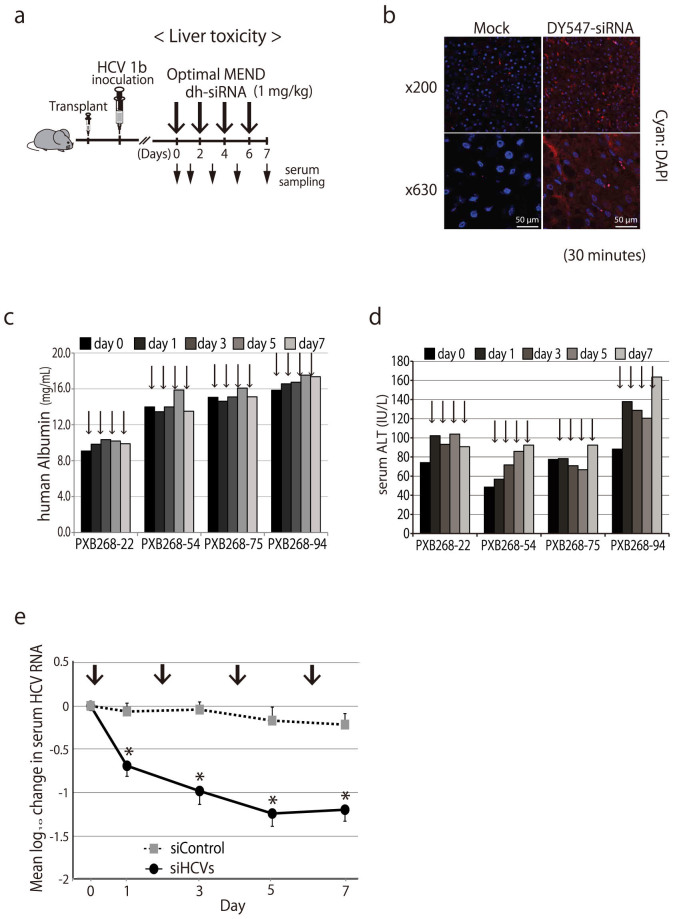
Liver toxicity of siRNA formulated in optimized MEND in humanized chimeric mouse. (a) Schedule of treatment by dh-siRNA formulated in optimized MENDs in chimeric mice carrying human hepatocytes (PXB mice) infected with HCV genotype 1b (HCR6). The mice were administered intravenously with siRNA-loaded optimized MENDs by 4 repeat doses. All optimized MENDs were loaded with siRNA and administered to provide doses of 1 mg/kg. (b) Distribution characteristics of siRNA in chimeric mice carrying human hepatocytes. DY-547-labeled siRNA formulated in optimized MENDs was injected intravenously into the orbital veins of chimeric mice. The liver was observed by fluorescence microscopy at 30 min after injection. The nuclei were stained with DAPI. Mock: unlabeled siRNA formulated in optimized MEND. (c, d) Liver toxicity by administration of siHCVs (the HCV-specific dh-siRNAs formulated in optimized MEND). Four-time injection of siRNA formulated in optimized MEND into HCV-infected chimeric mice (individual animals: PXB268-22, PXB268-54, PXB268-75, and PXB268-94) was performed on days 0, 2, 4, and 6 (indicated by vertical arrows). Serum human albumin levels (c) and alanine aminotransferase (ALT) levels (d) were monitored for 1 week. (e) Four-time injection of siHCVs and siControl into HCV-infected chimeric mice was performed on days 0, 2, 4, and 6 (indicated by vertical arrows). The HCV genomic RNA change from baseline following treatment with siHCVs (n = 4) or with siControl (non-targeting control siRNA formulated in optimized MEND, n = 3) were shown; data points are presented as the mean ± s.d.

We recently demonstrated the use of a novel mouse model for *in vivo* infection with hepatitis viruses^34^ (Fig. 4a). Specifically, the model consisted of severe combined immunodeficient (SCID) mice, transgenic for urokinase-type plasminogen activator (uPA), which were transplanted with human hepatocytes. (In the following text, this model will be referred to simply as “chimeric mice”.) We also have reported that these chimeric mice are a robust animal model to evaluate the efficacy of interferon and other anti-HCV agents^35^.

First, optimized MENDs encapsulating DY547-labeled siRNAs were administered intravenous (IV) to chimeric mice (Fig. 4b). After 30 minutes, accumulation of siRNA was observed in the livers of the chimeric mice. We noted that the intracellular distribution of siRNA was not patchy, but was instead diffusely organized in the cytoplasm, presumably reflecting endosomal escape following endocytosis^21^.

**Figure 5:**
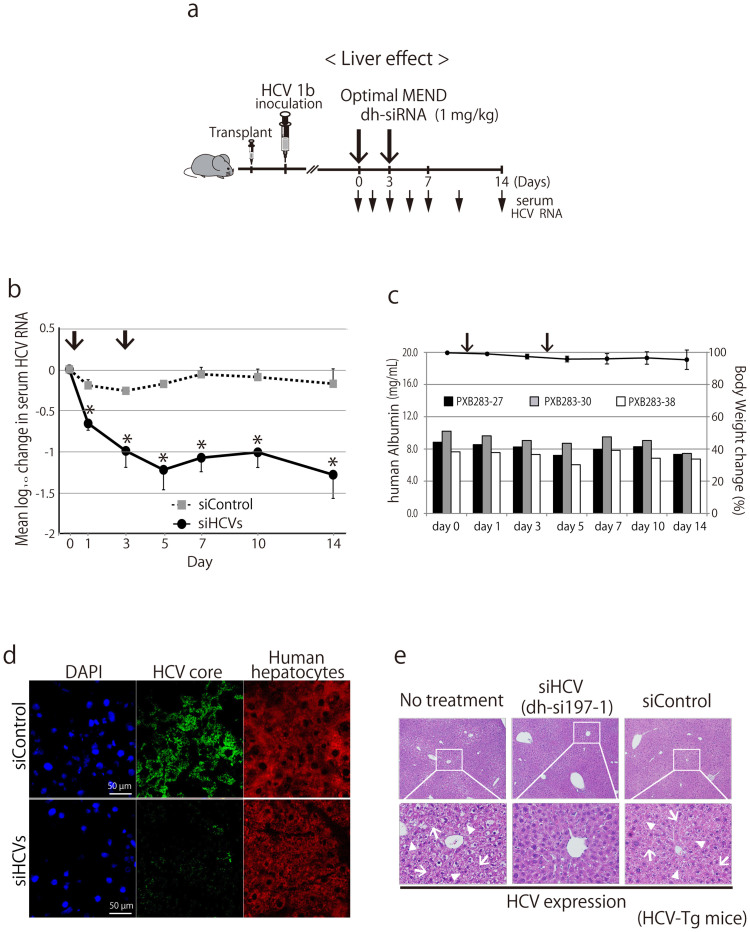
Silencing efficacy of siRNA formulated in optimized MEND against ongoing infectious HCV RNA. (a) Schedule of treatment by dh-siRNA formulated in optimized MENDs in chimeric mice carrying human hepatocytes (PXB mice) infected with HCV genotype 1b (HCR6). The mice were administered intravenously with siRNA (1 mg/kg)-loaded optimized MENDs by 2 repeat doses. (b) Long-term silencing efficacy of siHCVs (the HCV-specific dh-siRNAs formulated in optimized MEND) against ongoing infectious HCV RNA. The HCV genomic RNA change from baseline in individual mice following treatment with siHCVs (n = 3) or with siControl (n = 3) were monitored for 2 weeks. (c) The serum human albumin levels (indicated as bar in left y-axis) in individual animals, as well as the change of body weight (indicated as line in right y-axis; plotted as mean + s.d. (across 3 animals)) over 2 weeks. (d) Intrahepatic analysis of chimeric mice infected with HCV. Two weeks after administration of siHCVs or siControl (injected on day 0 and day 3), chimeric mouse liver was harvested. The presence of human hepatocytes and HCV core protein were evaluated by immunohistochemistry. (e) dh-siRNA-mediated amelioration of HCV-induced liver damage in a murine model of inducible HCV. The inducible-HCV transgenic mouse model (HCV-Tg mice; see Materials and Methods) provides conditional expression of HCV core, E1, E2, and NS2 proteins. Six months after HCV induction, mice were treated by injection (on days 0 and 2) with optimized MENDs loaded with a single species of dh-siRNA (si197-1). On day 4, livers were harvested and assessed histologically (hematoxylin and eosin staining) for HCV-induced liver inflammatory responses. Degenerated liver tissue with diffuse inflammation and spotty necrosis was observed in the livers of the “no treatment” and siControl mice; treatment with si197-1-loaded optimized MENDs reduced HCV-induced liver damage. Arrows indicate necrosis; arrowheads indicate inflammation.

Both the efficacy and tolerability of these dh-siRNA-loaded optimized MENDs are critical issues for potential human clinical usage. Therefore, we examined the toxicology (in the “humanized” livers of chimeric mice) of optimized MEND-siRNAs during repeat-dose administration. Specifically, dh-siRNAs were formulated in optimized MENDs and administered to chimeric mice for a total of four IV infusions (on days 0, 2, 4, and 6) at siRNA doses of 1 mg/kg (Fig. 4a). This treatment appeared to be well tolerated; human hepatocytes in the chimeric mice remained viable, as demonstrated by tracking of serum human albumin levels and serum alanine aminotransferase (ALT) levels (an indicator of liver toxicity) (Fig. 4c and 4d). Although a slight increase in ALT values was observed, the change was not additive over the course of successive infusions. (Fig. 4d). Consistent with the results, a mixture of the dh-si197-1, dh-si197-6, and dh-si50-10 (siHCVs) provided silencing of HCV replication (Fig. 4e). In contrast, a non-targeting control siRNA (siControl), also formulated in optimized MENDs, did not induce silencing of HCV replication (Fig. 4e). To evaluate the long-term efficacy of dh-siRNA-mediated silencing of HCV replication, we dosed chimeric mice with combined (three siRNAs; administered as IV infusions on days 0 and 3) and followed the animals for 2 weeks (Fig. 5a). As seen above for knockdown duration by systemic optimized MEND treatment (Fig. 3c), the combined siHCVs (dh-si197-1, dh-si197-6, and dh-si50-10, also formulated in optimized MENDs) suppressed HCV replication for 2 weeks (Fig. 5b); no adverse effects were seen in human serum albumin levels or body weights (Fig. 5c). At day 14, livers were also harvested and screened by immunohistochemistry (IHC) and for quantitation (via real-time RT-PCR) of hepatic HCV RNA. As shown in Figure 5d, intrahepatic HCV core protein (detected by IHC) was dramatically reduced by the siHCV treatment (PXB283-27 mouse) compared with the siControl treated mouse, in which HCV core protein was detected within human hepatocytes. RT-PCR^36^ demonstrated that the siHCV treatment provided an approximately 25-fold reduction in hepatic HCV RNA levels (treatment vs. siControl; 1.34 × 10^3^ vs. 3.36 × 10^4^ copies/μg total RNA, respectively).

Separate experiments, using another HCV pathogenesis mouse model^37^, demonstrated that siHCV (dh-si197-1) treatment improved HCV-induced liver inflammatory responses in conditional HCV transgenic mice (Fig. 5e).

## Discussion

The distinct effector phase of the RNAi pathway has been the focus of considerable research. This step depends on cleavage of the target mRNA by the RISC following base-pairing with the antisense strand of siRNA^38^. Therefore, the prediction of an effective siRNA had been evaluated based on the molecular mechanism of RISC assembly or the sequence preferences of the RISC endonuclease. Numerous strategies have been published to select siRNA sequences for targeting endogenous mRNA, and the resulting sequences have proved effective in some applications. However, the concept may not be appropriate to select target genes from other organisms, such as viruses.

Viruses typically exist as populations harboring multiple sequence variants, making these organisms notorious for the ability to develop resistance and escape control. Thus the prediction of effective antiviral siRNAs requires additional factors such as conservation of target sequence preferences. Based the level of sequence conservation among different HCV genotypes, the IRES of the 5′ UTR has been proposed as an RNAi target site. As highly conserved sequences are likely to contain structurally or functionally constrained elements, it has been argued that local higher order structures in target mRNAs might restrict accessibility to RISC, and attenuate or abolish RNAi activity. There are reports that the secondary structure of target sites in mRNAs strongly reduce siRNA-mediated RNAi activity^16^,^17^, hence the accessibility of certain local target structures on the mRNA is an important determinant in the gene silencing ability of siRNAs^18^. However, in general, RNA folding program such as mfold or sfold can predict more than one secondary structures for the same mRNA; it is difficult to know which of the proposed structures represents the real or the most frequent fold employed in the cell. Therefore, target mRNA structure is a criterion that cannot be easily defined nor confidently scored solely on the bases of *in silico* calculations.

To facilitate the prediction of highly active siRNA molecules in the HCV structurally elements, we used human Dicer endoribonuclease activity for preparation of certain siRNAs. Because RISC loading takes place in the context of the RISC-loading complex, which consists of an Argonaute protein, Dicer, and the dsRBD-containing protein TRPB in human cells^39^, Dicer favorable siRNA preference might be influence on loading to the complex. With mapping the corresponding cleavage site by the endoribonuclease-prepared siRNAs with the long dsRNA of IRES region, potent siRNA candidates in the structurally element have been identified by 5′ RACE methods. Thereby, we speculate that the accessibility of Dicer to target mRNA also may be a critical factor. Indeed, siRNA sequences derived by our hunting-by-Dicer method may be more potent inducers of RNAi than the siRNAs predicted by commercial design software.

The optimized MEND was accumulated in liver to around 90% of the injected dose within 30 min (data not shown). Recently, it was reported that neutral liposomal systems acquire ApoE in circulation, which enhances uptake of the liposomes into hepatocytes by low-density lipoprotein (LDL) receptor (LDLR) expressed on the surface of hepatocytes^40^,^41^. It also was reported that the average diameters of sinusoidal fenestrae in C57CL/B mice and healthy human are 141 nm and 107 nm, respectively^42^. The average diameter of optimized MENDs was around 80 nm (Fig. 3a), which would permit optimized MENDs to pass through fenestrae and access hepatocytes. Therefore, we assume that MENDs containing YSK05 are taken up by hepatocytes by a process mediated by ApoE-LDL receptor association. This association presumably follows extravasation of MENDs from sinusoidal lumen to Disse through fenestrae, which results in widespread delivery of siRNAs to hepatocytes in healthy and transgenic mice, as well as to human hepatocytes in chimeric mice. The lipid composition for the initial MEND *in vivo* was chosen at YSK05/DSPC/cholesterol/PEG-lipid = 50:10:40:3 mol%, as described previously^24^,^43^. Since DSPC, a helper lipid, was not essential to exert silencing in liver, DSPC was eliminated from lipid envelope (supplementary Fig. 2a). The pH-sensitive YSK05 lipid is presumed to be responsible for the endosomal release of the MEND cargo. Hence we tested the silencing activity of siRNA-loaded MENDs with increasing ratios of YSK05; activity was maximized at approximately 70 mol% YSK05. Based on our testing, a MEND composed of YSK05, cholesterol, and PEG-lipid at 70:30:3 was regarded as the optimized version. For systemic siRNA delivery using ionizable LNPs, lipid pKa value was identified as an important parameter, with optimum pKa in the range of 6.2–6.5^44^. In the present study, the pKa values of initial and optimized MENDs were determined as 6.6 and 6.4 respectively, which fell within this optimal pKa range. The endosomal escape of MEND is presumably mediated by membrane fusion. According to hemolysis activity, optimization of MEND improved fusion ability at acidic pH, but did not affect fusion at neutral pH. This optimization contributed to a 10-fold increase in silencing activity, yielding an ED50 of ~0.06 mg/kg in liver. Our results suggested that efficient siRNA delivery depends on the use of lipid-based nano-carriers that provide both optimized pKa and highly fusogenic characteristics. We also successfully delivered anti-miRNA oligonucleotide against miRNA-122 (miR-122) into hepatocytes using optimized YSK05-MEND, which resulted in efficient miR-122 knockdown and reduced plasma cholesterol level^45^,^46^. Our results permitted us to attempt delivery of dh-siRNAs to HCV-infected liver using the optimized MENDs. Our steady progress with a liver-targeting delivery system should facilitate the development of a safe and effective strategy for targeting HCV in hepatocytes in the near future.

Although our results were conducted in mouse models for HCV pathogenesis, results from these technologies are expected to provide therapeutic potential against infectious HCV *in vivo*, while also providing a new siRNA design tool for targeting viral seqeunces. Despite other obstacles (e.g., off-target effects), RNAi using these technologies provides a new potential therapeutic application that may effectively treat HCV infection.

## Methods

### Ethics statements

All *in vivo* experiments were approved by the Institutional Animal Care and Use Committee, and protocols for animal experiments were approved by the local ethics committee. The animals received humane care according to guidelines of the National Institutes of Health. Human patients provided informed written consent before sampling (collection of HCV-containing blood samples).

### siRNAs

The design of HCV-directed siRNAs has been described previously^26^. Briefly, we designed nine siRNAs that target the 5′-UTR and 3′-UTR of the HCV genome and examined their efficacy in the *in vitro* inhibition of HCV replication. Of these nine siRNAs, the most effective siE was one directed toward nucleotides 323 to 342 of the HCV genome. The sequences for the sense and antisense strands of the siRNA are as follows: siE sense: 5′-GUC UCG UAG ACC GUG CAU CAU U-3′; antisense: 5′-UGA UGC ACG GUC UAC GAG ACU U-3′. siRNAs were generated by annealing equimolar amounts of complementary sense and antisense strands.

Anti-luciferase siRNA (siLuc) (sense: 5′-CCG UCG UAU UCG UGA GCA ATT-3′; antisense: 5′-UUG CUC ACG AAU ACG ACG GTT-3′) was purchased from Sigma (Ishikari, Japan). Anti-FVII siRNA (siFVII) (sense: 5′-GGAucAucucAAGucuuAcT*T-3′; antisense: 5′-GuAAGAcuuGAGAuGAuccT*T-3′; lower case letters indicate 2′-fluoro-modified nucleotides, asterisks indicate phosphorothioate linkages) were purchased from Hokkaido System Science Co., Ltd. (Sapporo, Japan).

### Dicer-generated siRNAs

We generated the HCV-specific long dsRNA template for *in vitro* transcription by PCR-amplified DNA templates and synthesized Dicer-generated siRNAs (d-siRNAs) by cleavage with recombinant human Dicer (rhDicer; Gene Therapy Systems, San Diego, CA)^26^. By comparison with the silencing efficiency of d-siRNAs, d-siD5-50 and d-siD5-197 silenced the HCV replication more efficiently than synthetic siE (Supplementary information Fig. 1). The template dsRNAs of d-siD5-50 and d-siD5-197 were located at nucleotides 309–358 and 199-395 of HCR6 sequence (GenBank accession number AY045702).

### siRNAs predicted by the commercial software

The siRNA design algorithms for antiviral RNAi, siVirus, siDirect and Block-iT (Invitrogen), were used for the selection of the target sequence for siRNA within the specified target HCV genome (nucleotides 199–395).

### Cell culture and HCV-replicon assay

We used four HCV subgenomic replicon cell lines, FLR3-1 (genotype 1b, Con-1)^47^, R6FLR-N (genotype 1b, strain N)^26^, JFH-1/FLR/K4 (genotype 2a)^48^, and RMT-tri (genotype 1a)^49^, which have the firefly luciferase gene for the sensitive and precise quantification of the HCV replication levels using a luciferase assay. We also used REF cells^30^ which harbor the divided-full genome replicon for analysis of the HCV 5′ UTR sequences. Each cell line was seeded at a density of 5 × 10^3^ per well in 96-well tissue culture plates, and grown (at 37°C and 5% CO_2_) in complete Dulbecco's modified Eagle's medium supplemented with Glutamax I (Invitrogen, Carlsbad, CA) and containing 5% fetal calf serum (Invitrogen). Cells were transfected with 30 nM siRNA using RNAiMax (Invitrogen, Carlsbad, CA). After 72 hours, luciferase activity was determined in triplicate using the Steady-Glo or Bright-Glo luciferase assay kit (Promega Madison, WI). The luciferase signal was measured using an LB940 luminometer (Berthold, Freiburg, Germany) and the results were expressed as the mean percentage of control. IC_50_ values of siRNA were calculated by nonlinear curve-fitting using the equation: Y = 100-(Y_Bottom_ × X/(IC_50_ + X)), where Y represents percent inhibition and X represents the concentration of siRNAs.

### 5′-rapid amplification of cDNA ends (RACE) analysis

Replicon cells were transfected with synthetic HCV-specific siRNA (siE) and Dicer-generated siRNAs using Lipofectamine RNAiMAX or Lipofectamine 2000 (Invitrogen) according to the manufacturer's protocol. At 6 h post-transfection, total RNA from replicon cells was extracted using the acid guanidinium-phenol-chloroform method^26^.

For the RNA oligo ligation method, 5 μg of total RNA was ligated to the GeneRacer RNA adapter (Invitrogen, 5′-CGA CUG GAG CAC GAG GAC ACU GAC AUG GAC UGA AGG AGU AGA AA-3′) without any prior processing. Ligated RNA was reverse transcribed into cDNA using the HCV-specific reverse primer (R6 876-R20 reverse primer: 5′- AGA GGA AGA TAG AGA AAG AG-3′). To detect cleavage product, semi-nested-PCR was performed as follows: first-run PCR used a primer complementary to the RNA adapter (GeneRacer 5′ Nested Primer: 5′-GGA CAC TGA CAT GGA CTG AAG GAG TA-3′) in combination with an HCV-specific primer (R6 610-R24 reverse primer: 5′-CCC TCG TTG CCA TAG AGG GGC CAA-3′); second-run PCR used the same oligo specific primer (GeneRacer 5′ Nested Primer) in combination with a second HCV-specific primer (R6 536-R20 reverse primer: 5′-GAT AGG TTG TCG CCT TCC AC-3′).

For the C-tailing at the 3′-end of RNA method, first-strand cDNA synthesis was performed by using SuperScript II reverse transcriptase (Promega Corporation, Madison, WI) to transcribe total RNA (5 μg) with the HCV-specific reverse primer (R6 876-R20 reverse primer) according to the manufacturer's protocol. The resulting first-strand cDNA was subjected to another round of 5′-RACE using a distinct RACE kit (Cat. 18374-058, Invitrogen, Carlsbad, CA). The first-strand cDNA was tailed at the 3′-end by terminal transferase TdT and dCTP. The primer set consisted of an HCV-specific reverse primer (R6 610-R24) and the Abridged Anchor Primer for the first-run PCR, and an HCV-specific reverse primer (R6 536-R20) and the Abridged Universal Amplification Primer for the second-run PCR.

Amplification fragments obtained by the two independent 5′-RACE methods were resolved on 3.0% agarose and sized using a 1-kb Plus DNA Ladder (Invitrogen). Specific cleavage sites were further confirmed by DNA sequencing.

### Dicer-hunting siRNA sequences design

Based on the cleavage site defined by d-siRNAs, reverse genetic approach was applied to the design of Dicer favourable siRNA sequence. A dh-siRNA consists of duplexes of 21-nt RNAs that are base-paired with 2-nt 3′ overhangs.

### Preparation of MENDs

Cholesterol, 1,2-distearoyl-sn-3-phosphatidylcholine (DSPC), 1,2-dimyristoyl-sn-glycerol, and methoxyethyleneglycol 2000 ether (PEG-DMG) were purchased from Avanti Polar Lipid (Albaster, AL). The synthesis of YSK05 was performed as previously described^21^. MENDs encapsulating siRNAs were prepared by a *t*-BuOH dilution procedure. Lipid in 90% (v/v) *t*-BuOH was mixed with siRNA in 20 mM citrate buffer (pH 4.0) at siRNA/lipid ratio of 0.1 (wt/wt) under strong agitation to yield a final *t*-BuOH concentration of 60% (v/v). Then, the lipid/siRNA mixture was added into 20 mM citrate buffer (pH 4.0) under strong agitation to yield a final *t*-BuOH concentration of <12% (v/v). Ultrafiltration was performed to remove *t*-BuOH, replacing external buffer with phosphate-buffered saline (PBS, pH7.4) and concentrating the MENDs. A lipid envelope of initial MEND was prepared using YSK05, DSPC, cholesterol, and PEG-DMG at a molar ratio of 50:10:40:3 as described previously^24^,^43^, and optimized MEND was prepared using YSK05, cholesterol, and PEG-DMG at a molar ratio of 70:30:3.

### Characterization of MEND

The average diameter and zeta-potential of MENDs were determined using a Zetasizer Nano ZS ZEN3600 (MALVERN Instrument, Worcestershire, UK). siRNA encapsulation efficiency was determined by a RiboGreen assay (Invitrogen Carlsbad, CA). MENDs were diluted in 10 mM HEPES buffer (pH 7.4) containing 20 μg/mL dextran sulfate and Ribogreen in the presence or absence of 0.1% (w/v) Triton X-100. Fluorescence was measured by Varioskan Flash (Thermo scientific) with λex = 500 nm, λem = 525 nm. siRNA concentration was calculated based on a siRNA standard curve. siRNA encapsulation efficiency was calculated by comparing siRNA concentration in the presence and absence of Triton X-100. The pKa of YSK05 in each MEND was determined using 6-(p-Toluidino)-2-naphthalenesulfonic acid (TNS). Thirty μM of MEND lipid and 6 μM of TNS were mixed in 200 μL of 20 mM citrate buffer, 20 mM sodium phosphate buffer, or 20 mM Tris-HCl buffer, containing 130 mM NaCl at a pH ranging from 3.0 to 9.0. Fluorescence was measured by a Varioskan Flash with λex = 321 nm, λem = 447 nm, at 37°C. The pKa values were measured as the pH giving rise to half-maximal fluorescent intensity.

### Hemolysis assay

Fresh red blood cells (RBCs) were collected from ICR mice and suspended in PBS. The RBC suspension was mixed with indicated amount of MEND, incubated at 37°C for 30 min, and then centrifuged (4°C, 400 *g*, 5 min). The absorbance of the supernatant was measured at 545 nm. Positive and negative control samples were prepared by incubation of RBCs with 0.5% (wt/v) Triton X-100 or PBS (respectively). The %hemolysis was epressed as the % of the absorbance of the positive control.

### *In vivo* mouse Factor VII knockdown experiments

Male ICR mice (5–6 weeks old) were purchased from Japan SLC (Shizuoka, Japan). MENDs encapsulating siFVII were diluted to the appropriate concentrations in PBS (pH 7.4) and administered intravenously (IV; via the tail vein) at a dose volume of 10 to 15 mL/kg. At the indicated time points, blood and liver were collected. The blood was processed to plasma, and plasma levels of Factor VII protein were determined using a colorimetric Biophen VII assay kit (Aniara) according to the manufacturer's protocol. The standard curve for Factor VII plasma levels was generated using plasma collected from PBS-treated mice. Total RNA in liver was isolated using TRIzol (Invitrogen) according to the manufacturer's protocol. The resulting RNA was reverse transcribed using a High Capacity RNA-to-cDNA kit (ABI) according to manufacturer's protocol. For each specimen, quantitative PCR analysis was performed on 2 ng of cDNA using Fast SYBR Green Master Mix (ABI) and a Lightcycler480 system II (Roche). All reactions were performed in a volume of 15 μL. The primers for mouse *fvii* were (forward) 5′-TCG AAT CCA TGT CAG AAC GGA GGT -3 and (reverse) 5′-CCG ACC CTC AAA GTC TAG GAG GCA-3′.

### *In vivo* microscopic observation

Optimized MENDs encapsulating Cy5-labeled siRNA were administered into male ICR mice (5–6 weeks old). Five min before projected sacrifice, FITC-conjugated isolectin B4 (40 μg/mouse) was intravenously injected via the tail vein to stain blood vessels. At 30 min after intravenously injection of optimized MEND, each animal was perfused with PBS to remove blood from the liver, then with 4% paraformaldehyde (PFA)-PBS for fixation. Liver tissues were excised and further fixed with 4% PFA-PBS for 24 hr at 4°C, then submerged in 20% sucrose-PBS for 4 hr at 4°C. The liver was embedded in OCT compound (Sakura Fine Technical, Tokyo, Japan) and snap-frozen in liquid nitrogen. Frozen samples were cut in 30 μm-thick sections (LEICA CM3000, Leica Microsystems, Wetzlar, Germany). The samples were stained with Hoechst33342 to detect nuclear DNA, and observed at an excitation wavelength of 633 nm using a laser-equipped Nikon A1 (Nikon Co. Ltd., Tokyo, Japan) with a x60 objective lens.

### *In vivo* HCV infection experiments

We purchased chimeric mice from PhenixBio (Hiroshima, Japan). The chimeric mice were generated by transplanting human primary hepatocytes into severe combined immunodeficient (SCID) mice carrying the urokinase plasminogen activator transgene controlled by an albumin promoter (uPA/SCID)^34^. Six weeks after hepatocyte transplantation, each mouse was injected IV with patient serum containing 10^6^ copies of HCV genotype 1b (HCR6; accession number AY045702)^36^. HCV inoculations, drug administration, blood collection, and sacrifice were performed under ether anesthesia. Blood samples were taken from the orbital vein and sera were immediately isolated. Human serum albumin in the blood of chimeric mice was measured with a commercially available kit (Alb-II kit; Eiken Chemical, Tokyo, Japan) and serum ALT level was determined with enzymatic assays (Horiba ABX Diagnostics) according to the manufacturer's instructions^35^.

Rhodamine-labeled siRNA was synthesized by Dharmacon (Lafayette, CO). Alexa-546 or Alexa-568 labeled siE/CL-LA was injected IV into BALB/c mice. After 30 minutes, the liver, lung, spleen, and kidney were harvested from each mouse. Sections of these tissues then were stained with DAPI (Molecular Probes) and slides examined using confocal laser microscopy (Zeiss).

Liver tissues obtained from mice were embedded in OCT compound (Ted Pella, Redding, CA). The frozen tissues were cut into thin sections (6 μm) and placed on glass slides. The sections were fixed in 10% buffered formalin and then treated with 0.1% Triton X-100. To detect HCV protein by immunohistochemistry (IHC)^35^, the slides were incubated with rabbit anti-core protein IgG and then with donkey anti-rabbit IgG polyclonal antibody (Fab fragment, labeled with HRP; Dako, Glostrup, Denmark). The HRP label was amplified with FITC-conjugated tyramide according to the manufacturer's instructions (Molecular Probes, Eugene, OR). To detect human hepatocytes, liver sections were probed with anti-human hepatocyte monoclonal antibody (Dako), followed by anti-mouse IgG-Alexa 546 (Molecular Probes). Nuclei were stained by DAPI. Normal rabbit IgG was used as a control.

### Transgenic mice with persistent HCV protein expression

To provide an immunocompetent model for inhibition of HCV protein expression, a mouse strain harbouring an HCV transgene was generated via a Cre/loxP switching system^37^. We bred CN2-29 transgenic mice, which carry an HCV transgene (nt. 294–3435), with Mx1-Cre transgenic mice, which express Cre recombinase in response to interferon (IFN)-α or a chemical inducer of IFN-α, poly(I:C). Following poly(I:C) injection, the HCV transgene was rearranged, and HCV sequences were expressed in the livers. In this model, HCV structure proteins are expressed in the liver within 7 days after poly(I:C) injection. Male CN2-29 trasgegenic mice (8–9 week-old) were injected intraperitoneally with 0.3 mL of 1 mg/mL poly(I:C) solution [in PBS (-)]. At 6 months after the poly(I:C) injection, the CN2-29 mice were administrated intravenously twice with the siRNA-MEND complex solution [1 mg/mL in PBS(-)] via orbital sinus at day 0 and day 2. The mice were sacrifice under anesthesia with ketamine/xylazine 2 days after the second siRNA-MEND administration. Livers were removed, fixed in 10% buffered formalin, and embedded in paraffin. Section (4 μm) were stained with hematoxylin and eosin, and observed using ZEISS Axio Imager A2 upright microscope (Carl Zeiss MicroImaging, Inc, Germany).

### Statistical analysis

The data are expressed as the mean ± S.D. Statistical analysis of the difference between the HCV viral load during treatment and follow-up period and the baseline (day 0) level was conducted using the analysis of variance with a nonparametric Mann-Whitney U test. The probability values P < 0.05 were marked with *, and P < 0.01 were marked with **.

## Author Contributions

M.K. conceived the study. T.W., H.H., C.M. and Y.S. conducted the study equally. T.W. and H.H. coordinated the analysis and manuscript preparation. M.S. and H.H. had input into the study design and A.T., Y.H. and T.O. accomplished mouse management. M.A. and K.I. revised the manuscript for intellectual content. T.W., H.H., C.M. and Y.S. contributed equally.

## Supplementary Material

Supplementary Information Supplementary Information
