# Supplementary material for: In vivo therapeutic potential of Dicer-hunting siRNAs targeting infectious hepatitis C virus
Source: Sci Rep. 2014 Apr 23;4:4750. doi: 10.1038/srep04750 (PMC3996463; doi:10.1038/srep04750)
Supplement: Supplementary Information [file srep04750-s1.doc]

**Supplementary information**

***In vivo* therapeutic potential of Dicer-hunting siRNAs targeting infectious hepatitis C virus.**

Tsunamasa Watanabe^1, 2, 6^†, Hiroto Hatakeyama^3^†, Chiho Matsuda-Yasui^1^†, Yusuke Sato^3^†, Masayuki Sudoh^4^, Asako Takagi^1^, Yuichi Hirata^1, 2^, Takahiro Ohtsuki^1^, Masaaki Arai^5^, Kazuaki Inoue^2^, Hideyoshi Harashima^3^ and Michinori Kohara^1,*^

“nt No.” corresponds to: full length HCV genome (GenBank accession number AY045702).

**
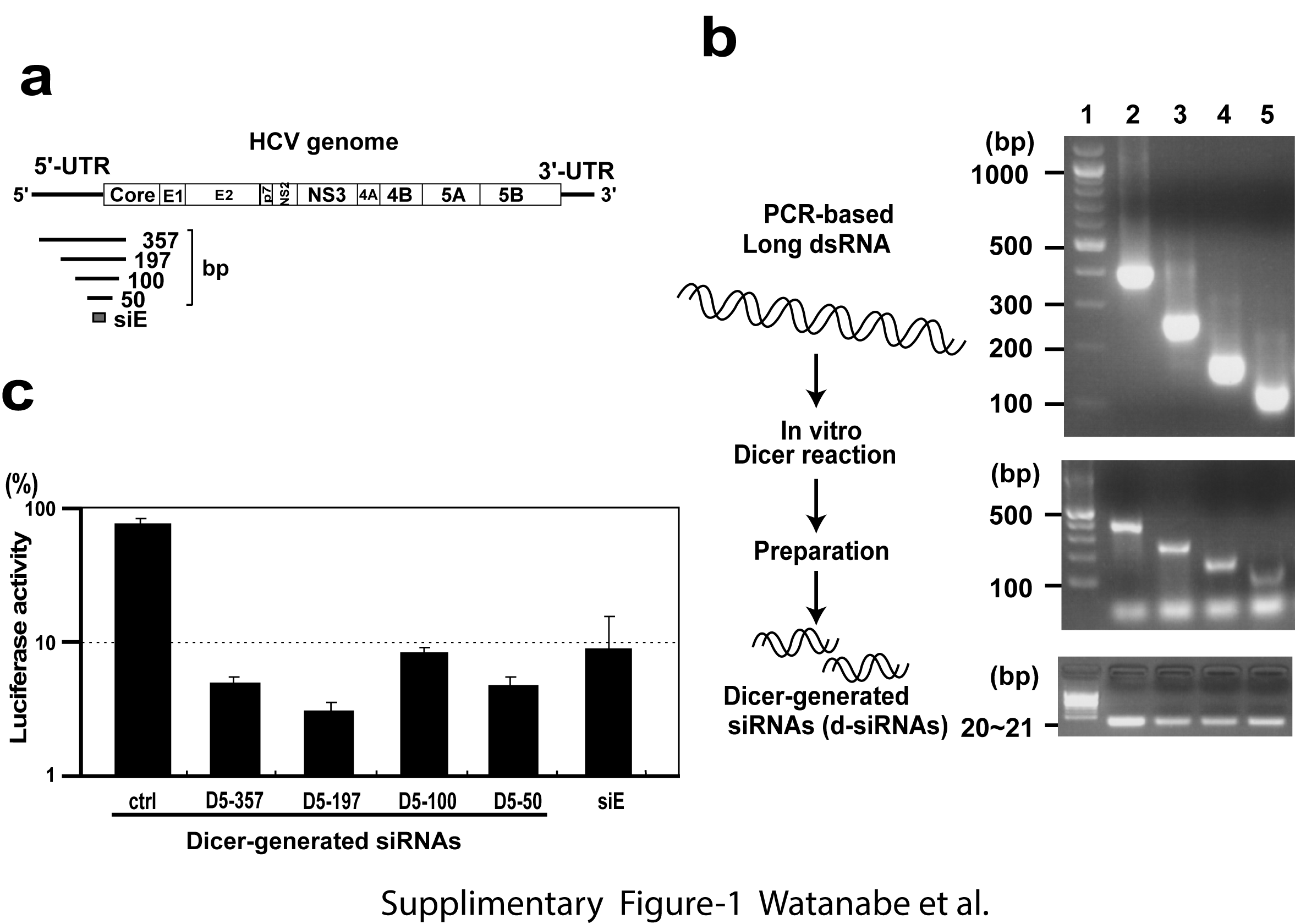
**

**Supplementary Figure 1. Dicer-generated siRNA synthesis**

**Supplementary Figure 1 materials and methods**

HCV-specific long dsRNAs were prepared by *in vitro* transcription of PCR-amplified DNA templates. A modified T7 promoter sequence was added to the 5’-end of each PCR primer for amplification. The dsRNAs were produced from the purified DNA templates using an Ampliscribe T7 transcription kit (Epicenter Technologies, Madison, Wisconsin). Single-stranded RNA was converted to double-stranded RNA by allowing annealing of the two strands. The dsRNA was purified and then digested with recombinant human Dicer (rhDicer; Gene Therapy Systems, San Diego, CA) according to the manufacturer’s protocol. The resulting Dicer-generated siRNAs (d-siRNAs) were separated by electrophoresis on a nondenaturing 12% polyacrylamide gel and detected by ultraviolet shadowing on a fluor-coated thin-layer chromatography plate (Ambion). The d-siRNAs migrating as 20- to 21-bp bands were excised from the gel and extracted at 37 ºC for 4 h in extraction buffer (0.5 M ammonium acetate, 1 mM EDTA, and 0.2% SDS). Following buffer exchange and desalting by gel filtration with Sephadex G-25 (Amersham Biosciences, Piscataway, NJ), the d-siRNAs were dissolved in TE buffer (10 mM Tris-HCl, 1 mM EDTA, pH 8.0), quantified by absorbance at 260 nm, and stored at –80 ºC.

**Supplementary Figure 1 legend**

**a)** Schematic representation of the long dsRNAs used for targeting different sites in the HCV genome RNA. Alignment shows relative positions of the synthetic HCV-directed siRNAs (sized as indicated) and the siE RNA.

**b)** Schematic outline of the synthesis of Dicer-generated siRNAs (d-siRNAs). The d-siRNAs were generated from the long HCV-specific dsRNAs by cleavage with rhDicer *in vitro*. Lane 1, 100-bp ladder DNA marker; Lane 2, targeting 357-bp dsRNA; Lane 3, targeting 197-bp dsRNA; Lane 4, targeting 100-bp dsRNA; Lane 5, targeting 50-bp dsRNA. Top to bottom, the three panels of B show fluorescence on non-denaturing gels of the originating material, the Dicer-digested material, and the final product.

**c)** HCV sub-genomic replicon (R6FLR-N; genotype 1b strain)[^1^](#_ENREF_1) cells were transfected with d-siRNAs. Luciferase activity was measured 48 h after transfection with 1 nM siRNA or d-siRNA. Data are presented as mean ± s.d. (n=3) of values normalized to the activity in mock-transfected cells. Transfections were performed with the following reagents: Ctrl, control d-siRNAs targeting p53 mRNA (766 bp); D5-357, d-siRNAs generated from the 357-bp dsRNA; D5-197, d-siRNAs generated from the 197-bp dsRNA; D5-100, d-siRNAs generated from the 100-bp dsRNA; D5-50, d-siRNAs generated from the 50-bp dsRNA.


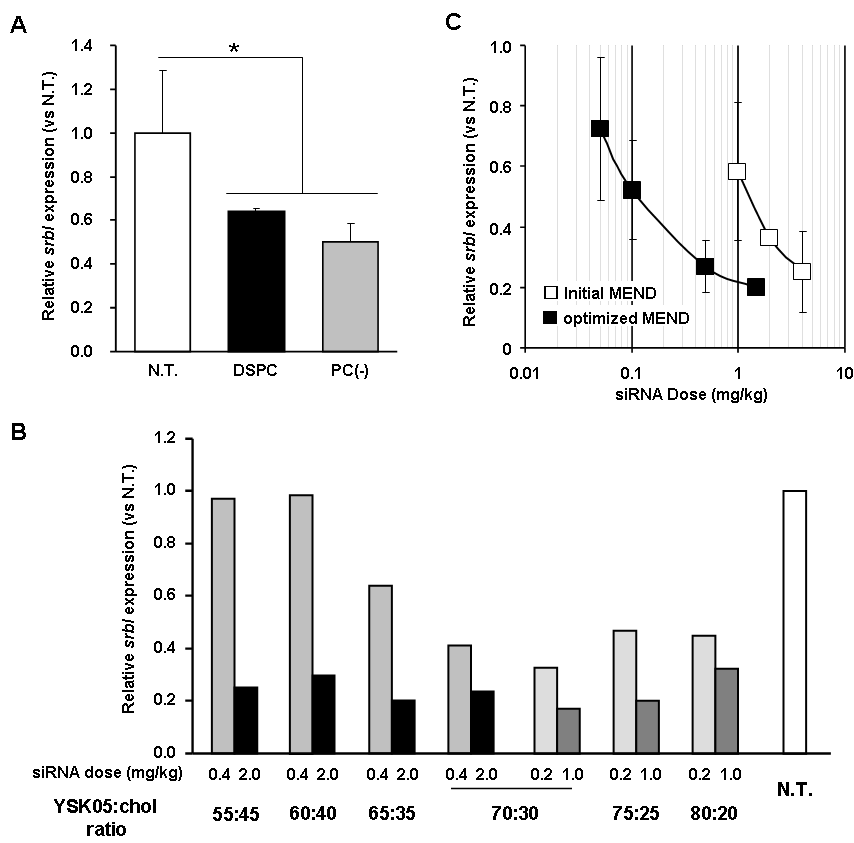


**Supplementary Figure 2. Optimization of MEND containing YSK05**

**Supplementary Figure 2 materials and methods**

Optimization was performed by varying lipid composition. MENDs were used to encapsulate an anti-*srbI* siRNA consisting of two oligonucleotides: 5’- guc gca ugg cuc aga gag uTT -3’ (sense strand) and 5’- acu cuc uga gcc aug cga cTT -3’ (anti-sense strand), where the upper- and lower-case letters indicate DNA and RNA, respectively. The resulting particles were administered intravenously to ICR mice (5-6 weeks olds) at a dose of 1.5 mg/kg (panel A) or at the indicated doses of siRNA (panels B and C). At 24 hr after injection, liver was collected and total RNA was isolated using TRIzol (Invitrogen) according to the manufacturer’s protocol. Resulting RNA was reverse transcribed using a High Capacity RNA-to-cDNA kit (ABI) according to the manufacturer‘s protocol. A quantitative PCR analysis (15 μL per reaction) was performed on 2 ng of cDNA using Fast SYBR Green Master Mix (ABI) and the Lightcycler480 system II (Roche). The primers for mouse *srbI* were as follows: forward, 5’-AAT AAA GGC TTG GAG AAC CC-3’; reverse, 5’-ACC TCA CCT GTC TCT CGA AC-3’. The primers for mouse *gapdh* were as follows: forward, 5’- AGC AAG GAC ACT GAG CAA G -3’; reverse, 5’- TAG GCC CCT CCT GTT ATT ATG -3’.

**Supplementary Figure 2 legend**

**a)** Initial screen for improvement of efficacy of MENDs by varying lipid composition. A lipid envelope of MEND was initially prepared using YSK05, DSPC, cholesterol, and PEG-DMG at a molar ratio of 50:10:40:3 (denoted in the panel as DSPC). A lipid envelope lacking the DSPC component was prepared by combining YSK05, cholesterol, and PEG-DMG at a molar ratio of 60:40:3 (denoted in the panel as PC(-)). Mice (n=3 per group) received no treatment (N.T.) or were injected intravenously (at 1.5 mg/kg) with anti-*srbI* siRNA encapsulated in DSPC or PC(-) MENDs. Livers were collected 24 hr later; resulting RNA was reverse transcribed and used for quantitative PCR of *srbI* expression (compared to housekeeping gene *gapdh*). Data are presented as mean ± sd (n=3) of values normalized to the N.T. value. **P*<0.05. Because the knockdown efficacy in liver was phospholipid-independent, further experiments were performed using MENDs lacking phospholipid.

**b)** Knockdown efficacy of MENDs containing varying proportions of YSK05. The YSK05 fraction in the lipid envelope was altered from 55 to 80 mol%. MENDs containing 70 mol% of YSK05 exhibited the largest knockdown of target *srbI* gene expression in liver. Based on these results, MENDs composed of YSK05/cholesterol/PEG-DMG (70:30:3) were further tested as the “optimized MEND”.

**c)** Dose-response curves comparing the knockdown efficacy of initial MEND (YSK05:DSPC:cholesterol:PEG-DMG = 50:10:40:3) and optimized MEND (YSK05:cholesterol:PEG-DMG = 70:30:3) in liver. The dose-response curves show that ED50s of initial and optimized MENDs were approximately 1-2 mg/kg and 0.1 mg/kg, respectively. Thus, the optimization of MEND composition improved efficacy by approximately 1 order of magnitude. Data are presented as mean ± s.d. (n=3).

**Supplementary References**

1. Watanabe, *T., et a*l. Intracellular-diced dsRNA has enhanced efficacy for silencing HCV RNA and overcomes variation in the viral genotype*. Gene thera*py 13, 883-892 (2006).
